# Fastest Formation Routes of Nanocarbons in Solution Plasma Processes

**DOI:** 10.1038/srep36880

**Published:** 2016-11-14

**Authors:** Tetsunori Morishita, Tomonaga Ueno, Gasidit Panomsuwan, Junko Hieda, Akihito Yoshida, Maria Antoaneta Bratescu, Nagahiro Saito

**Affiliations:** 1 Department of Material Science and Engineering, Graduate School of Engineering, Nagoya University, Furo-cho, Chikusa-ku, Nagoya, 464-8603, Japan; 2 NU- PPC Plasma Chemical Technology Center, The Petroleum and Petrochemical College, Chulalongkorn University, Bangkok 10330, Thailand; 3 CREST, JST, Furo-cho, Chikusa-ku, Nagoya, 464-8603, Japan

## Abstract

Although solution-plasma processing enables room-temperature synthesis of nanocarbons, the underlying mechanisms are not well understood. We investigated the routes of solution-plasma-induced nanocarbon formation from hexane, hexadecane, cyclohexane, and benzene. The synthesis rate from benzene was the highest. However, the nanocarbons from linear molecules were more crystalline than those from ring molecules. Linear molecules decomposed into shorter olefins, whereas ring molecules were reconstructed in the plasma. In the saturated ring molecules, C–H dissociation proceeded, followed by conversion into unsaturated ring molecules. However, unsaturated ring molecules were directly polymerized through cation radicals, such as benzene radical cation, and were converted into two- and three-ring molecules at the plasma–solution interface. The nanocarbons from linear molecules were synthesized in plasma from small molecules such as C_2_ under heat; the obtained products were the same as those obtained via pyrolysis synthesis. Conversely, the nanocarbons obtained from ring molecules were directly synthesized through an intermediate, such as benzene radical cation, at the interface between plasma and solution, resulting in the same products as those obtained via polymerization. These two different reaction fields provide a reasonable explanation for the fastest synthesis rate observed in the case of benzene.

Liquid-phase plasma^1^,^2^,^3^,^4^,^5^ has attracted the attention of several researchers engaged in plasma science, especially for applications involving materials and water treatment^6^,^7^,^8^,^9^,^10^. The properties of liquid-phase and atmospheric plasmas differ even though liquid-phase plasma is a particular type of atmospheric plasma. Several types of liquid-phase plasma, e.g., streamer, spark, arc, and glow discharge plasmas, and their various formation methods have been reported^11^,^12^,^13^,^14^. We have already demonstrated a spark–glow transition plasma in aqueous solution and have used it to fabricate gold nanoparticles in the absence of a reducing agent^15^,^16^,^17^,^18^,^19^,^20^,^21^,^22^,^23^,^24^. This plasma is named solution plasma. It is a non-equilibrium plasma in solution, and the characteristics of plasma and solution are coupled by the exchange of electrons and ions.

Solution plasma can potentially be used for the precise nanomaterial synthesis. In general, the electron temperature is higher than the ion temperature in the case of non-equilibrium plasma^25^. However, the temperature at the center of the plasma is still high, i.e., approximately 4000 K^26^,^27^, even though the solution temperature is maintained at approximately room temperature. Thus, a temperature gradient exists between the center of the plasma and the plasma–solution interface. This temperature gradient is extremely large because the plasma is surrounded by liquid as a condensed phase. The activated particles, including ions, electrons, radicals, and photons, are quenched and then deactivated. However, such species have sufficient energy to induce precise chemical reactions but not physical changes. Unique reactions can thereby occur at the interface under room temperature. Various researchers have used the solution plasma to synthesize Au, Pt^28^, AuPt^29^, Pd^30^, PdAu^31^, Ag^32^, Au alloys^33^,^34^, metal oxide nanoparticles^35^,^36^,^37^,^38^, and nanosheets^39^,^40^,^41^. It has also been used to modify the surface of carbon materials to achieve monodispersion^42^, to prepare metal nanoparticles on carbon^43^,^44^,^45^,^46^, and to degrade gelatin^47^, among other applications^48^,^49^,^50^,^51^,^52^,^53^,^54^,^55^,^56^,^57^,^58^,^59^.

Recently, we attempted to adapt the solution plasma system to organic solutions (see Supplementary video and Fig. 1S). In such systems, the plasma enables unique reactions such as C–H activation reactions at the interface between the solution and plasma, whereas conventional plasma induces random decomposition reactions. We have fabricated graphene, heterographene, nanocarbon sheets, and nanocarbon spheres via solution plasma from organic solutions containing solvents such as benzene, toluene, pyridine, or pyrazine^60^,^61^,^62^,^63^,^64^,^65^,^66^,^67^,^68^,^69^,^70^,^71^,^72^. Moreover, the reaction rate is substantially higher than the rates of other synthesis methods such as chemical vapor deposition and pyrolysis synthesis. However, solution plasma is a relatively new process and the knowledge accumulated thus far is insufficient to enable the design and control of the processes and products.

There are two reaction routes from solution, i.e., routes from starting substances to products. The first is the reaction route through plasma, which is composed of the vaporized solution and decomposed species and where the gas temperature approaches 4000 K. The second is the reaction route at the interface between the plasma and solution, where the ion temperature is as low as room temperature, but the electron temperature is still sufficient to advance the organic reaction. Nanocarbons might form at this interface because the structure and composition of the product are inherited from the starting substances. If the reaction in plasma is the main route, the product structure and composition will become random and complex. However, the details of reaction mechanism are not known.

In this study, the nanocarbon formation routes via the solution plasma process were investigated. To simplify the reaction routes for synthesizing nanocarbons, we used starting materials categorized as either linear or ring molecules. The products were analyzed by gas chromatography–mass spectrometry (GC/MS), X-ray diffraction (XRD), Raman spectrometry, and *ab initio* MO calculations. The intermediates involved in the conversion of the starting materials to nanocarbon products were estimated; the nanocarbon formation routes are discussed.

## Results and Discussion

**Figure 1:**
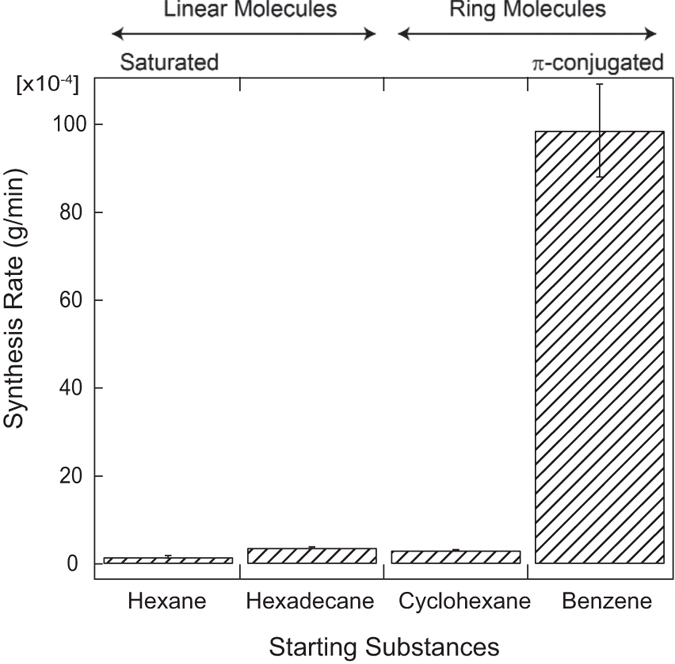
Synthesis rates of nanocarbons from hexane, hexadecane, cyclohexane, and benzene.

Nanocarbons were synthesized using solution plasma from hexane, hexadecane, cyclohexane, and benzene. The synthesized nanocarbons were collected, and the synthesis rates of the nanocarbons were estimated. Figure 1 shows the synthesis rates of nanocarbons from hexane, hexadecane, cyclohexane, and benzene. The respective synthesis rates of nanocarbons were 1.6 × 10^−4^, 3.7 × 10^−4^, 3.1 × 10^−4^, and 98.6 × 10^−4^ g/min. The synthesis rates of nanocarbons obtained from linear molecules, i.e., hexane and hexadecane, were obviously lower than that of nanocarbons obtained from ring molecules, i.e., benzene. In particular, the synthesis rate of nanocarbon from benzene was substantially greater than that of nanocarbons from the other investigated solvent. This result means that the π-conjugated bonds in a ring structure enhanced nanocarbon synthesis in the case of solution plasma.

**Figure 2:**
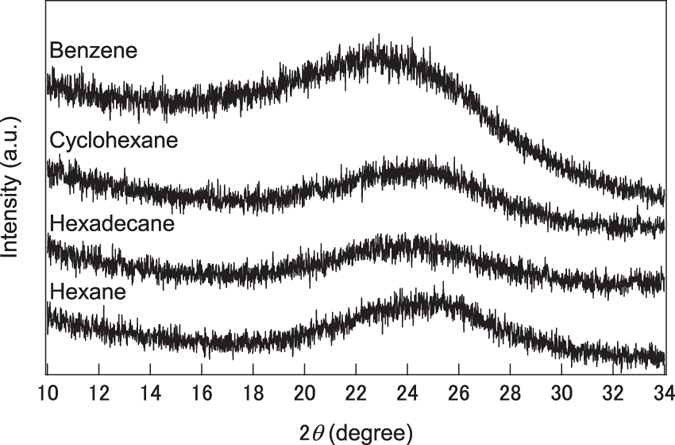
XRD patterns of the nanocarbons obtained from hexane, hexadecane, cyclohexane, and benzene.

**Figure 3:**
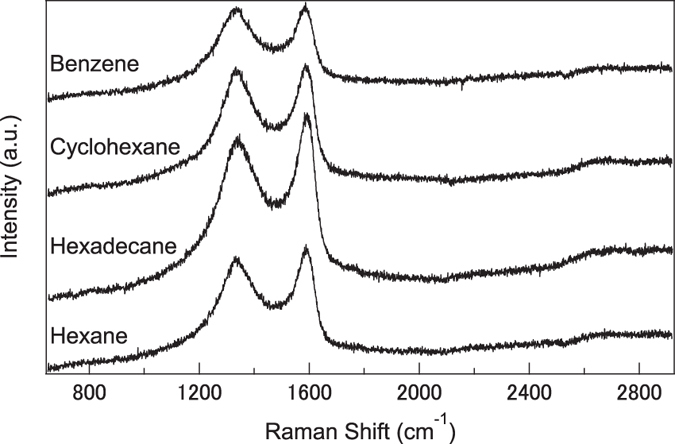
Raman spectra of the nanocarbons obtained from hexane and benzene.

To characterize the carbon structure synthesized from linear and ring molecules, XRD and Raman spectra of the hexane, hexadecane, cyclohexane, and benzene systems were collected; the results are shown in Figs 2 and 3. The XRD patterns of all nanocarbons show broad peaks around 2*θ* = 24° corresponding to the (002) plane of graphite. These peaks are different by the molecule and the calculated lattice spacing of hexane, hexadecane, cyclohexane, and benzene were 0.357, 0.371, 0.365, and 0.386 nm, respectively. Lattice spacing of hexane is closer to that of highly oriented pyrolytic graphite. It means the crystallinity of nanocarbon synthesized from hexane is higher than that from benzene. In Raman spectra, the D- and G-bands appeared at 1350 and 1600 cm^−1^, respectively. The D-band originates from the mixing of sp^3^ orbitals in a plane with structural defects and/or impurities, whereas the G-band originates from the graphite structure. The ratio between the intensities of the D- and G-bands, *I*(D)/*I*(G), is a crystallinity index for graphite because *I*(D)/*I*(G) = 0 for single-crystalline graphite. The *I*(D)/*I*(G) ratio for the nanocarbon prepared from hexane and hexadecane were 0.933 and 0.894, whereas that for the nanocarbon prepared from cyclohexane and benzene were 0.978 and 0.984. Thus, the crystallinity of the nanocarbons synthesized from linear molecules is greater than that of nanocarbons synthesized from the corresponding ring molecules, although the synthesis rate of nanocarbons obtained from π-conjugated ring molecules is higher than that of nanocarbons prepared from linear molecules.

**Figure 4:**
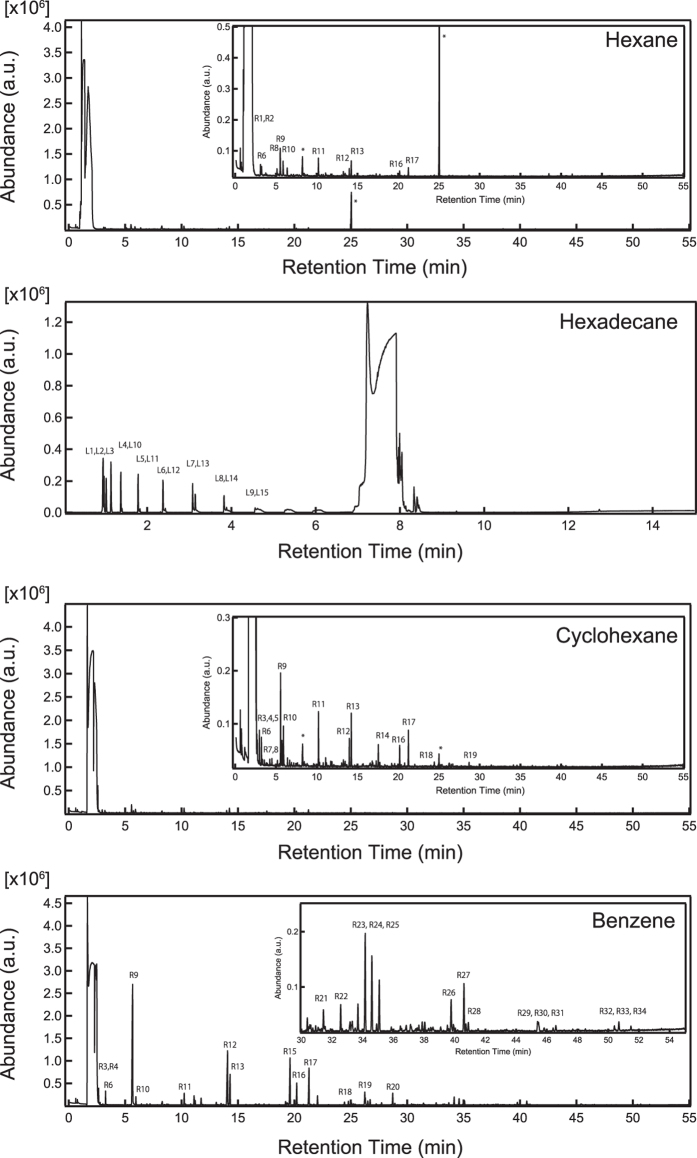
GC/MS qualitative analysis of the products synthesized from hexane, hexadecane, cyclohexane, and benzene. The peak labels correspond to the labels in Figs 5 and 6.

**Figure 5:**
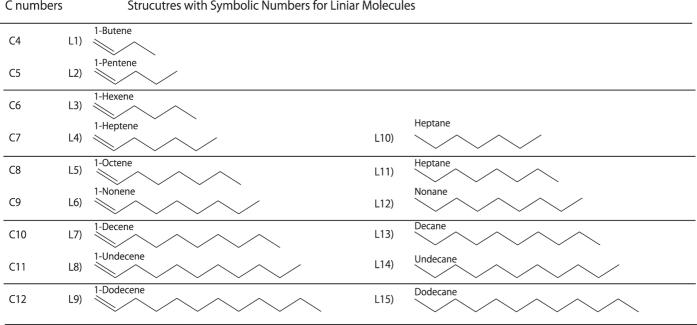
The products obtained from the linear structures with symbolic numbers corresponding to the GC/MS spectrograms in Fig. 4.

**Figure 6:**
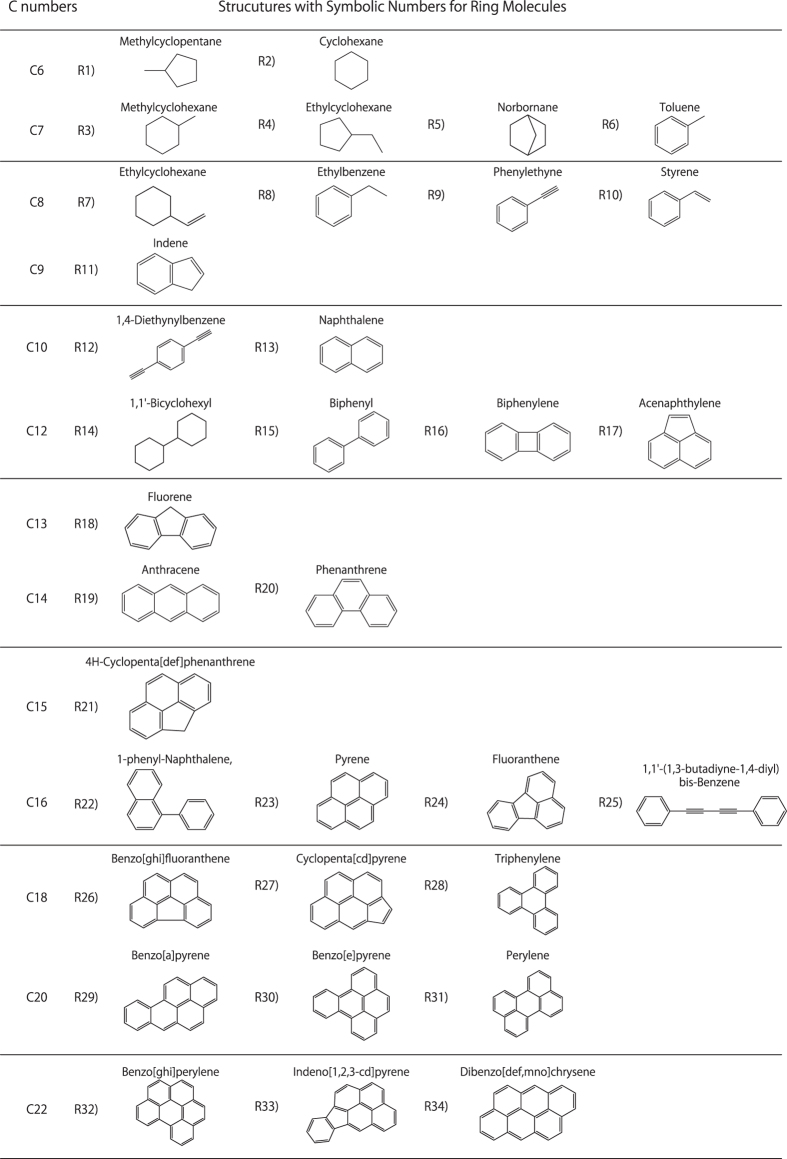
The products obtained from the ring structures with symbolic numbers corresponding to the GC/MS spectrograms in Fig. 4.

Figure 4 shows the GC/MS qualitative analysis results for the products synthesized from hexane, hexadecane, cyclohexane, and benzene. Figures 5 and 6 list the products of the linear and ring structures, with symbolic numbers L1)–L15) and R1)–R34) corresponding to the peaks in the GC/MS spectrogram. The products from the linear molecules were as follows. The products synthesized from hexane were R1), R2), R6), R8)~R13), R16) and R17). The products synthesized from hexadecane were L1)~L15). The products from the ring molecules were as follows. The products synthesized from cyclohexane were R3)~R14) and R16)~R19). Finally, the products synthesized from benzene were R3), R4), R6), R9)~R13) and R15)~R34).

Among the products of the syntheses starting from the linear molecules, small linear molecules and one- or two-ring molecules were observed. Specifically, molecules with one or two rings appeared in the product obtained from hexane, and the parts of rings molecules tend to be converted into π-conjugated ring molecules. Meanwhile, shorter olefins were observed in the product obtained from hexadecane. The peak intensities of the shorter olefins were greater than those of the longer ones. This product tendency was attributed to byproducts by the plasma decomposition. Oppositely, one- to six-ring molecules appeared in the products obtained from the synthesis of the ring molecules. A comparison of the peak intensities reveals that one-ring molecules tend to be directly converted into two- and/or three-ring molecules, not converted through smaller olefins.

**Figure 7:**
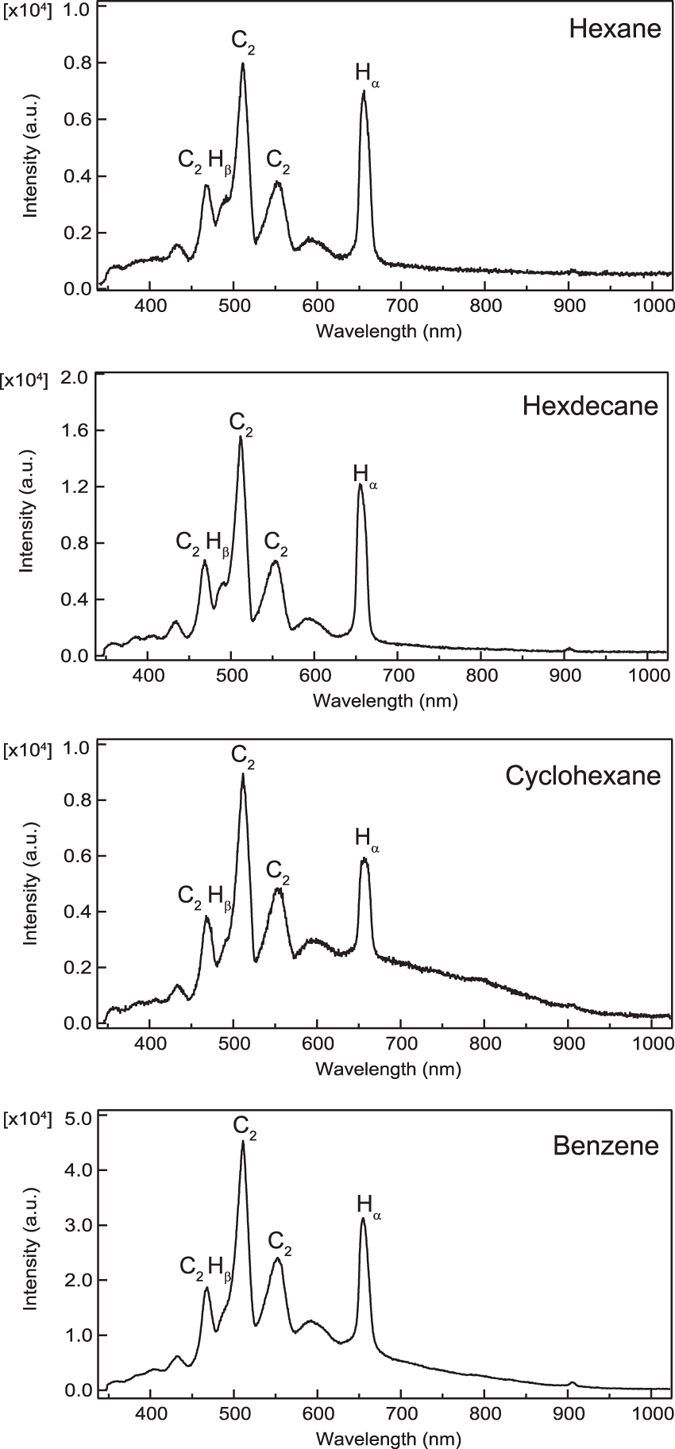
Optical emission spectra for plasmas formed in hexane, hexadecane, cyclohexane, and benzene.

Optical emission spectroscopy (OES) was performed to elucidate the relation between the states of plasma and the obtained products. Figure 7 shows the OES spectra for plasma formed from hexane, hexadecane, cyclohexane, and benzene. For all OES spectra, H_α_, H_β_, and C_2_ lines were observed. The ratios of the peak intensities were approximately the same. Moreover, the background shapes were roughly the same. These results indicate that the plasma temperature was approximately uniform at the plasma center. From the blackbody radiation approximation, the plasma temperature at the center was approximately 4000–5000 K, according to Wien’s displacement law. Such temperatures are sufficient to form graphite via organic compound decomposition.

**Figure 8:**
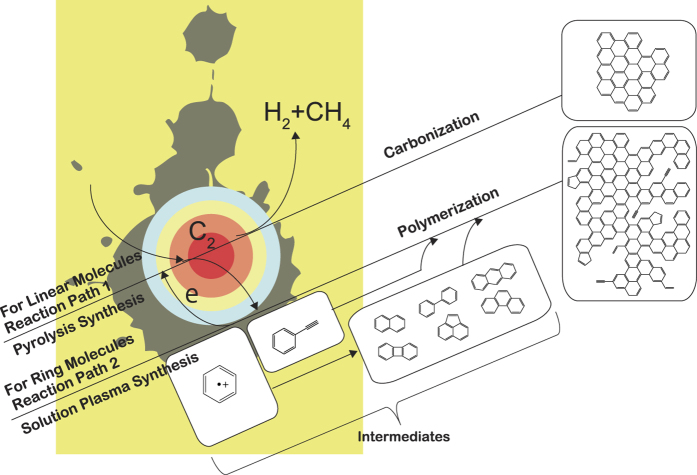
Reaction routes from hexane, hexadecane, cyclohexane, and benzene.

Two reaction paths from monomer to polycyclic aromatic hydrocarbons (PAHs), including graphene, are shown in Fig. 8. One reaction path (reaction path 1) is from the plasma center. The solution is vaporized by a strong electric field between electrodes. The vaporized solution forms a gas phase between the electrodes, and the gas phase is converted into plasma after breakdown. In the plasma, organic compounds were almost completely decomposed, similar to the products obtained via pyrolysis synthesis. Meanwhile, the OES results indicate that H atoms and C_2_ molecules were the main products at the plasma center. C_2_ molecules undergo association reactions, i.e., carbonization, under the higher temperatures of approximately 4000–5000 K. Finally, the overall reaction provides graphite, graphene, and carbon-related materials^73^. By contrast, C_2_ molecules were generated at the interface between the plasma and solution. The temperature around the interface remained closer to the solution temperature. These C_2_ molecules might exist as C_2_H_2_ and/or C_2_H_4_ because hydrogenation can occur at such temperatures. These molecules react with the main molecules into the solution—in our case, hexane, hexadecane, cyclohexane, and benzene—and the main molecules are associated or polymerized. The other reaction route is through the interface between the plasma and solution, where the temperature is sufficiently low. Non-equilibrium plasma in the liquid phase, such as solution plasma, requires secondary electrons to maintain the plasma state because it lacks electrons. In general, secondary electrons are supplied from electrodes, as they are frequently composed of metals. In the case of solution plasma, the solution component also functions as electrodes (i.e., liquid electrodes)^1^,^2^,^4^,^5^,^74^,^75^, enabling the plasma to gain secondary electrons from the solution, i.e., molecules. Upon the emission of the secondary electrons, radical cations form into the solution. In the case of the solution where the formation of this radical cation preferentially occurs, this reaction path (reaction path 2) is favored over reaction path 1 because the volume of plasma is small. In the reaction path 2, the organic compounds and/or monomer are polymerized without the decomposition involved in the reaction path 1.

When the linear molecules were used as starting materials, the synthesis rates were smaller than those of ring molecules and the products were more crystalline than those of ring molecules. These results indicate that the nanocarbon products obtained from the linear molecules are synthesized through the plasma center, which results in a greater crystallinity of the products compared to those obtained from the ring molecules. The *I*(D)/*I*(G) ratios in the Raman spectra of the products obtained from the linear molecules such as hexane are lower than those of the products obtained from the ring molecules, although the ratios of the products obtained from linear molecules slightly vary. When the ring molecules were used as starting materials, the synthesis rate was higher than that when the linear molecules were used. The reaction rate has been particularly fast in the case of benzene. The expected intermediate, benzene radical cations, easily formed because of the presence of delocalized unsaturated bonds, i.e., π-conjugated orbitals. This benzene radical cation is known to be an important intermediate to polycyclic aromatic hydrocarbon (PAH)^76^,^77^,^78^,^79^,^80^,^81^,^82^,^83^,^84^,^85^,^86^. The solution plasma can induce benzene radical cation formation even though this formation typically requires dedicated catalysts.

**Figure 9:**
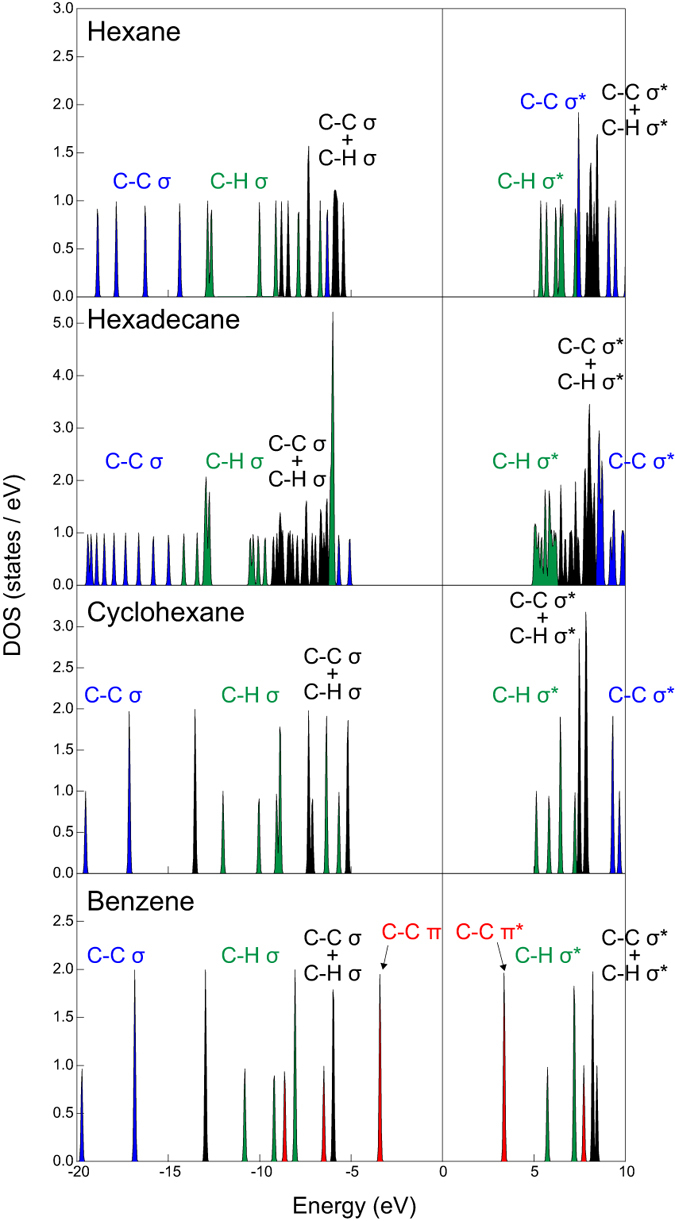
The density of states of hexane, hexadecane, cyclohexane, and benzene. The respective states are divided according to the contribution type of C–C σ, C–C σ*, C–C π, C–C π*, C–H σ, and C–H σ* orbitals.

To discuss the formation of radical cations, we predicted the density of states of hexane, hexadecane, cyclohexane, and benzene using *ab initio* MO calculations, as shown in Fig. 9 (and Fig. 2S).

In Fig. 9, π-bonding and π-antibonding orbitals, σ-bonding and σ-antibonding orbitals of C–H, and σ-bonding and σ-antibonding orbitals of C–C are described by their respective contributions. The calculation results indicate that in the case of the linear molecules, the C–C bonds and C–H bonds will be decomposed in the plasma—not at the interface—because they are strongly stabilized. Specifically, the highest occupied molecular orbital (HOMO) and lowest unoccupied molecular orbital (LUMO) of hexane indicate that C–H dissociation and/or C–H and C–C dissociation at the tail end of chains are the primary reactions (see Fig. 2S). The GC/MS results for the product obtained from hexane show that the primary reactions proceed via thermal reactions in the plasma at the early stage of the following reactions (1)–(6):


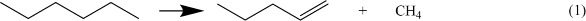


In the case of hexadecane, the HOMO and LUMO indicate that the primary reactions of C–C dissociation of at the tail end, followed by C–H dissociation occur as secondary reactions (see Fig. 2S). In the case of hexadecane, shorter olefins were produced, whereas ring molecules were not observed. A longer discharge treatment will produce ring molecules such as cyclohexane. The HOMO and LUMO of cyclohexane indicate that C–H dissociation proceeds as the primary reaction, which leads to unsaturated ring molecules such as benzene (see Fig. 2S). Furthermore, activated carbon materials function as catalysts for the dehydrogenation reaction of cyclohexane^87^. In our reaction system, the synthesized carbons can enhance the following dehydrogenation reaction:


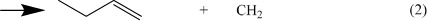



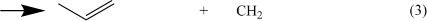



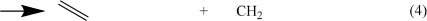



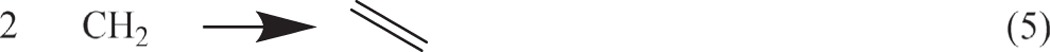



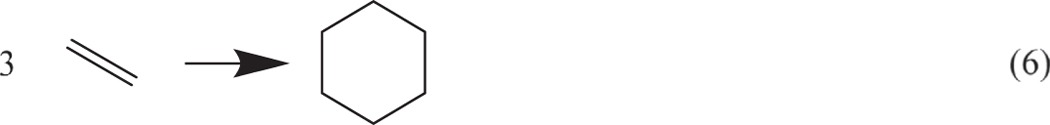



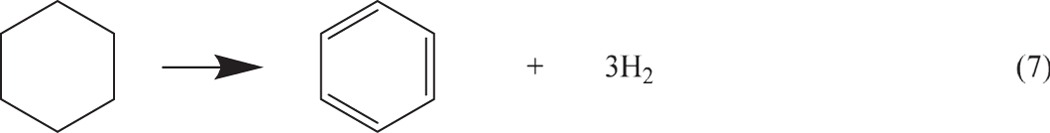


Oppositely, in the case of benzene, π-conjugated bonding and antibonding orbitals are closer to the Fermi level, i.e., benzene exhibits greater reactivity. The electrons in π- orbitals are easily excited to π* orbitals because of the plasma sheath potential, which is the same as the electrical double-layer potential in electrochemical reactions. Furthermore, the excited electrons are emitted by collisions of particles, including ions and electrons, at the interface. Benzene radical cations will be formed at the interface through the excitation and emission of electrons (see the reaction (8)). These cations are considered an important intermediate in the formation of PAHs, i.e., nanocarbons. Benzene radical cations are converted into biphenyl and then into triphenylene as the following via reactions (8)–(11):


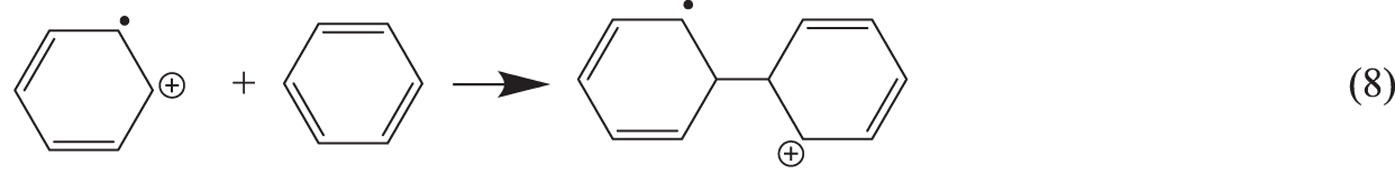



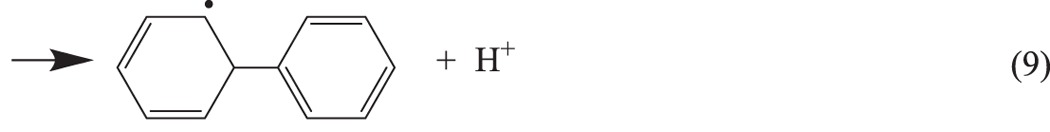



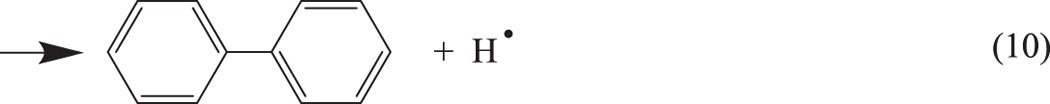



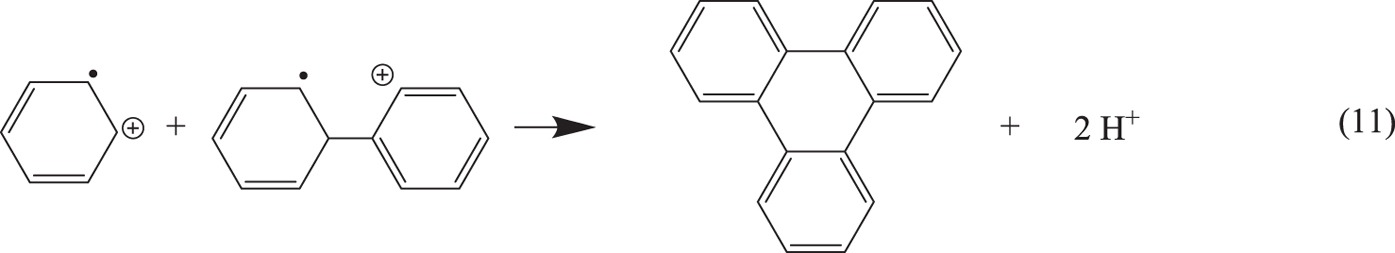


In general, the polymerization rate of benzene radical cations is fast; the benzene radical cations are continuously produced at the plasma–solution interface because of the plasma sheath. Moreover, the benzene radical cation is converted into *o*-benzyne and then into biphenylene through reactions (12) and (13):


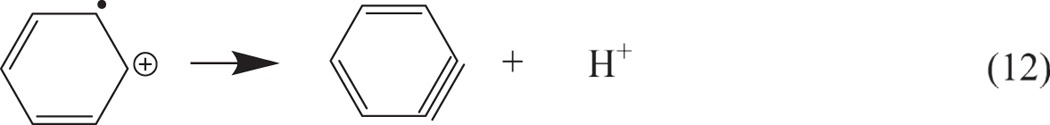



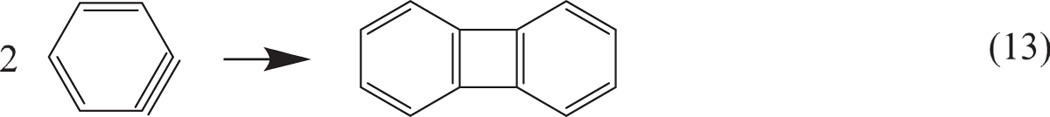


As previously discussed, the two reaction fields (one in the plasma and the other at the interface) can explain the higher synthesis rate of nanocarbons achieved in the case of benzene.

## Conclusion

In this study, we investigated nanocarbon formation routes from hexane, hexadecane, cyclohexane, and benzene via solution plasma. To simplify the possible routes, starting materials categorized as linear or ring molecules were used. GC/MS analysis revealed that the linear molecules were decomposed into shorter olefins, whereas the ring molecules were reconstructed. Saturated ring molecules were polymerized through cation radicals, such as benzene radical cations, and were converted into two- and three-ring molecules. In comparison to nanocarbons obtained from the ring molecules, nanocarbons obtained from the linear molecules exhibited greater crystallinity. This difference is a consequence of different reaction routes. The nanocarbons obtained from linear molecules were synthesized from small molecules, starting from C_2_ molecules, through the plasma region—where they were exposed to heat—similar to the pyrolysis synthesis process. By contrast, the nanocarbons obtained from the ring molecules were synthesized through intermediates, such as the benzene radical cations, at the plasma–solution interface, similar to the mechanism encountered in the polymerization. Because of these different routes, the crystallinity of the nanocarbons obtained from the ring molecules was lower than that of the nanocarbons obtained from the linear molecules. To confirm the schemes above, the reactivities of the starting materials were evaluated via *ab initio* MO calculations. In the case of the linear molecules, the primary and secondary reactions are the decomposition of C–C bonds and C–H bonds, respectively. Conversely, in the case of the ring molecules, the primary reaction is the excitation of π-bonds. The secondary reaction is the emission of electrons from molecules, which leads to the formation of cation radicals, which serve as the intermediate to PAHs.

## Methods

**Figure 10:**
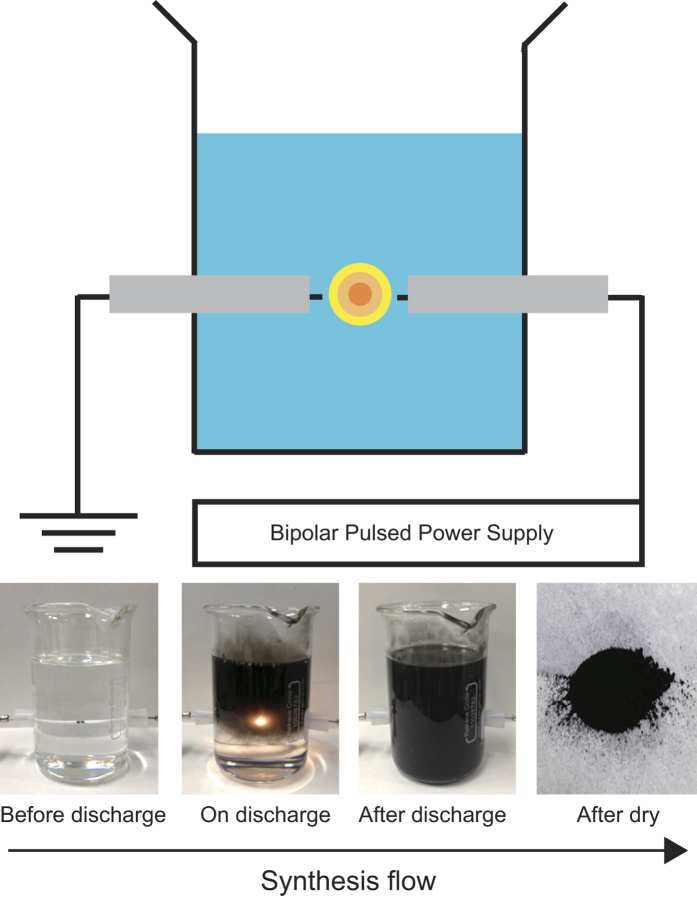
Schematic of the experimental setup for solution plasma experiments and synthesis flow for nanocarbon production.

Carbon nanomaterial samples were prepared using a solution plasma process. Figure 10 shows the experimental setup for the solution plasma and synthesis flow of nanocarbons. A bipolar pulsed power supply (Kurita Co. Ltd., MPS-R06K01C-WP1-6CH) was used to form the solution plasma. The electrodes were tungsten wires with 1 mm diameter. The electrodes were covered with isolated segments of ceramic. The applied peak voltage between the electrodes was about 1700 V, the repetition frequency was 15 kHz, the pulse width was 1.0 μs, and the electrode gap was 0.5 mm. Plasma was characterized by OES using a spectrograph (Ocean Optics Co. Ltd., USB2000+).

The following chemicals were used as starting substances: hexane (≥99%; Sigma-Aldrich Co. Ltd.), hexadecane (>98.0%; Kanto Kagaku Co. Ltd.), cyclohexane (>99.5%; Kanto Kagaku Co. Ltd.), and benzene (>99%; Kanto Kagaku Co. Ltd.). These chemicals were used as received for carbon nanomaterials synthesis.

After the synthesis of the carbon nanomaterials by solution plasma processes, the excess liquids were removed via suction filtration through a membrane filter composed of hydrophilic PTFE having a pore diameter of 0.1 μm (Merck Millipore Co. Ltd., Omnipore JVWP04700). The samples were then completely dried at 100 °C in an oven (Yamato Co. Ltd., DX301). The amounts of carbon obtained from each solution with a 250 mL volume during 20 min were measured using a balance (AS-ONE Co. Ltd., EK2000i).

X-ray diffraction (XRD) patterns were recorded using an X-ray diffractometer (Rigaku Co. Ltd., Smartlab) with Cu Kα radiation (λ = 0.15406 nm) operating at 45 kV and 200 mA. Raman spectra were recorded on a spectrometer (JASCO Co. Ltd., NRS-1000) with a laser excitation wavelength of 532 nm.

The intermediate products were confirmed by GC/MS (JEOL JMS-Q1050GC) analysis; the instrument was equipped with a 30-m HP-5 column (Agilent Technologies 19091J-413). Helium was used as a carrier gas for GC at a flow rate of 1.4 mL/min. The column temperature was varied from 40 °C to 300 °C at a temperature ramp rate of 5 °C/min to 20 °C/min. Mass spectrometry was performed for the mass-to-charge ratio range from 20 to 600 *m*/*z*. The 1-μL samples were syringed by the splitless injection method at an inlet temperature 350 °C. Hexane, cyclohexane, and benzene samples were concentrated to ten times by using an oven at 85 °C after the treatment.

To investigate the reactivity of the starting materials, we conducted density functional theory calculations using the Gaussian09 software package^88^. B3LYP was applied as an exchange-correlation functional. The basis set adopted for C and H was 6–31G*.

## Additional Information

**How to cite this article**: Morishita, T. *et al.* Fastest Formation Routes of Nanocarbons in Solution Plasma Processes. *Sci. Rep.* **6**, 36880; doi: 10.1038/srep36880 (2016).

**Publisher’s note:** Springer Nature remains neutral with regard to jurisdictional claims in published maps and institutional affiliations.

## Supplementary Material

Supplementary Information

Supplementary Video 1
