# Supplementary material for: Fastest Formation Routes of Nanocarbons in Solution Plasma Processes
Source: Sci Rep. 2016 Nov 14;6:36880. doi: 10.1038/srep36880 (PMC5107960; doi:10.1038/srep36880)
Supplement: Supplementary Information [file srep36880-s1.pdf]

Fastest Formation Routes of Nanocarbons in Solution Plasma Processes

Tetsunori MORISHITA[1], Tomonaga UENO[1,2,3], Gasidit PANOMSUWAN[2], Junko HIEDA[1], Akihito YOSHIDA[1], Maria Antoaneta BRATESCU[1], Nagahiro SAITO[1,2,3*]

1) Department of Material Science and Engineering, Graduate School of Engineering, Nagoya University, Furo-cho, Chikusa-ku, Nagoya, 464-8603, Japan

2) NU- PPC Plasma Chemical Technology Center, The Petroleum and Petrochemical College, Chulalongkorn University, Bangkok 10330, Thailand

3) CREST, JST, Furo-cho, Chikusa-ku, Nagoya, 464-8603, Japan

*Corresponding author: hiro@rd.numse.nagoya-u.ac.jp

Supplementary:

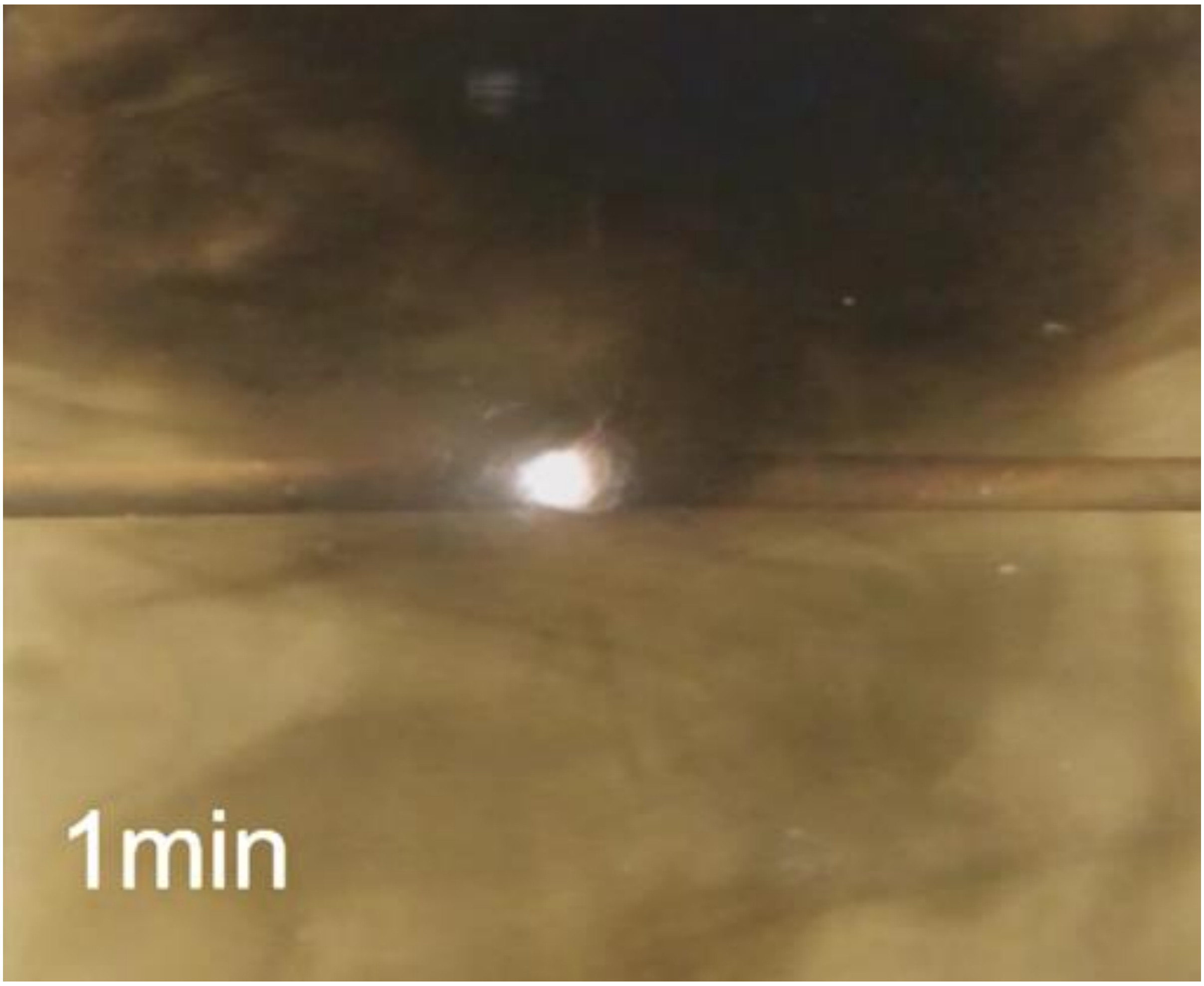

Supplementary V. A video file showing the nanocarbon synthesized from benzene (see Supplementary information_movie.mov). The black matter is nanocarbons (i.e., graphene, carbon sheets, carbon spheres). When the plasma conditions and/or solution were changed, the shapes were varied.

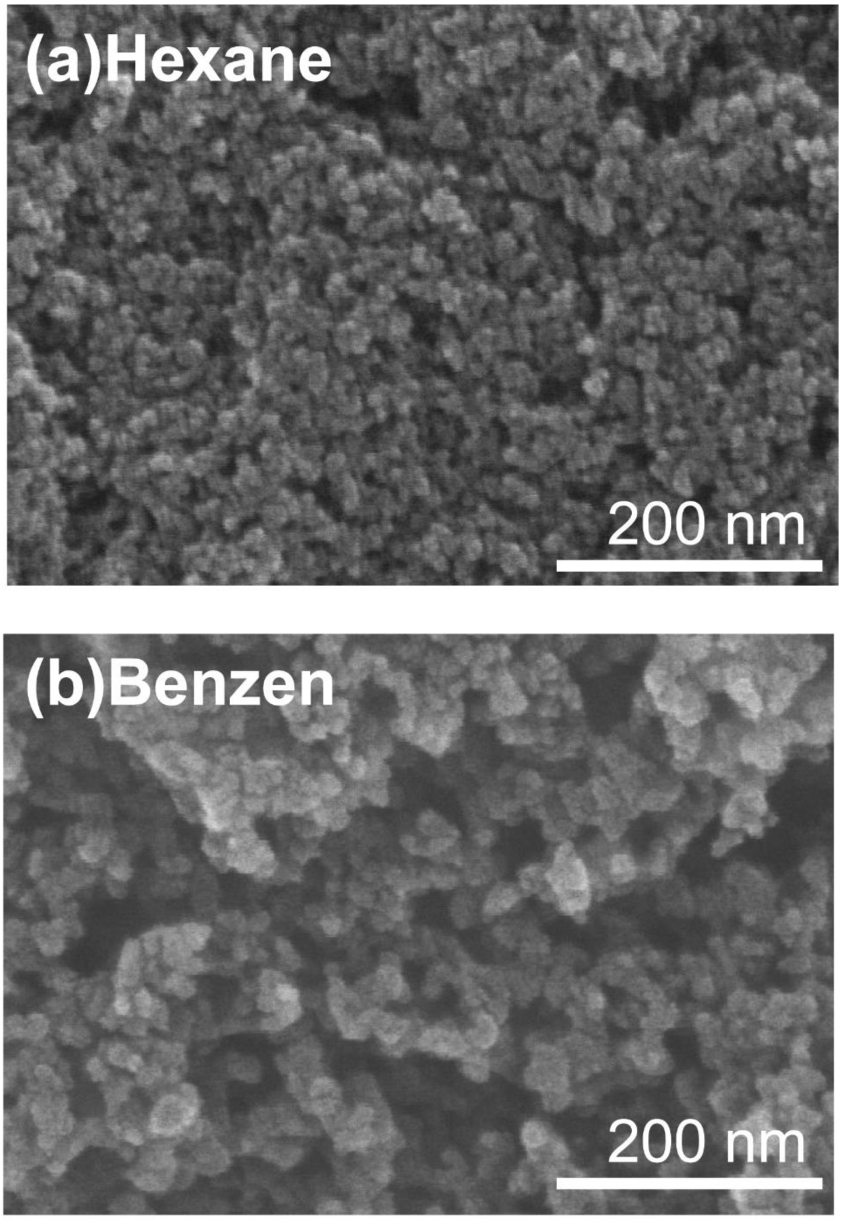

Fig. 1S FE-SEM images of nanocarbons obtained from (a) hexane and (b) benzene. The nanocarbon synthesized from benzene contained larger spheres, whereas the nanocarbon synthesized from hexane contained smaller spheres, similar to nanocarbons obtained through pyrolysis synthesis.

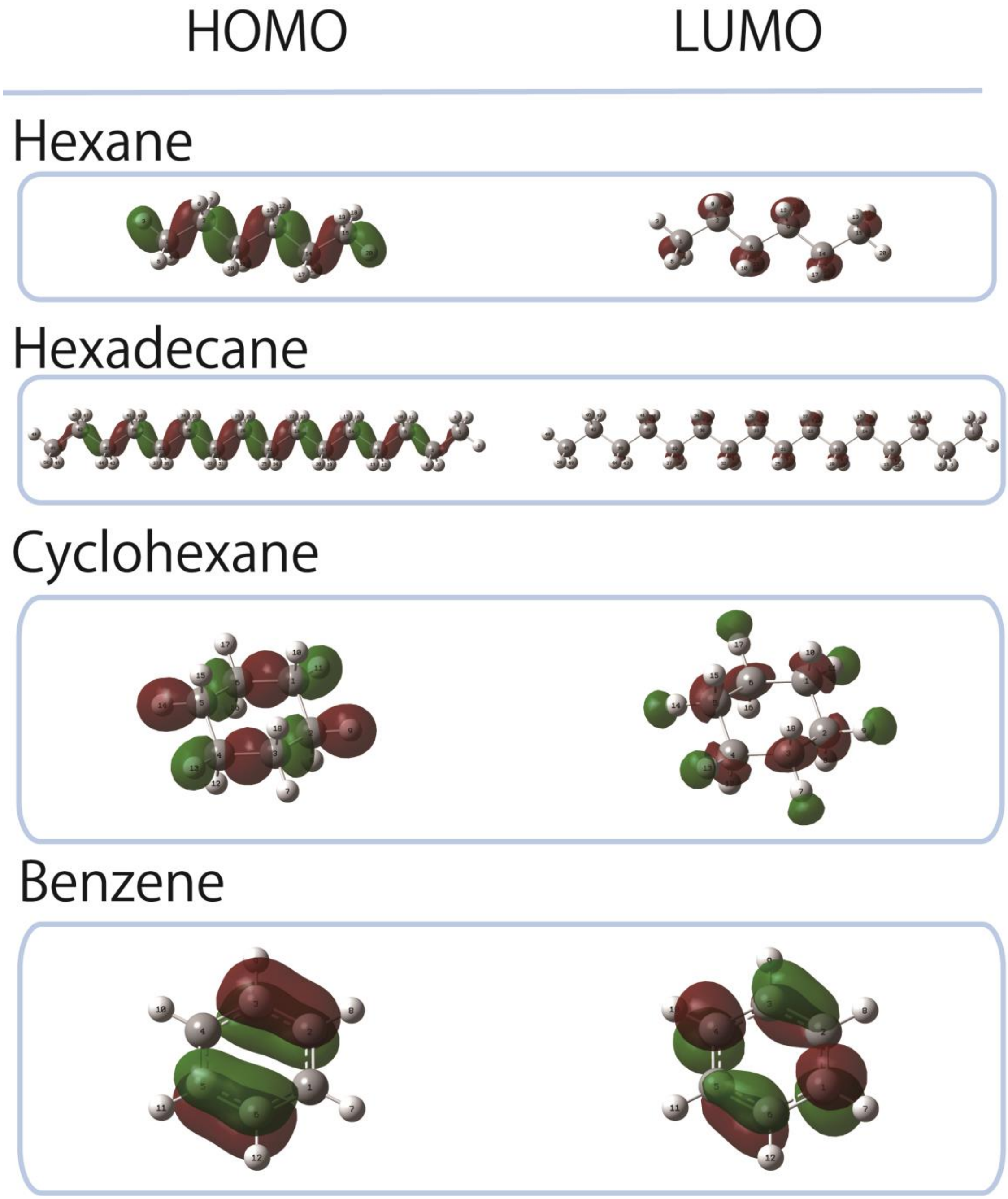

Fig. 2S Highest occupied molecular orbital and lowest unoccupied molecular orbital of hexane, hexadecane, cyclohexane, and benzene.
